# Description and Outcomes of an Ultrasound-Guided Technique for Catheter Placement in the Canine Quadratus Lumborum Plane: A Cadaveric Tomographic Study and Clinical Case Series

**DOI:** 10.3390/vetsci11100472

**Published:** 2024-10-02

**Authors:** Massimiliano Degani, Géraldine Bolen, Chiara Talarico, Stéphanie Noël, Kris Gommeren, Chiara Di Franco, Charlotte Sandersen

**Affiliations:** 1 Department of Clinical Sciences, Faculty of Veterinary Medicine, Fundamental and Applied Research for Animals & Health (FARAH), University of Liège, 4000 Liège, Belgium; gbolen@uliege.be (G.B.); ctalarico@uliege.be (C.T.); stephanie.noel@uliege.be (S.N.); kris.gommeren@uliege.be (K.G.); charlotte.sandersen@uliege.be (C.S.); 2 Department of Veterinary Sciences, University of Pisa, 56122 Pisa, Italy; chiara.difranco@phd.unipi.it

**Keywords:** analgesia, dog, quadratus lumborum block, computed tomography, postoperative period, locoregional anesthesia

## Abstract

**Simple Summary:**

In human medicine, catheter implantation for local anesthetic administration in the quadratus lumborum (QL) inter-fascial plane (IFP) has been reported to extend the duration of a single-shot block’s effect in the postoperative period of patients undergoing abdominal surgeries. In the first phase of this study, we described an ultrasound-guided technique for placing catheters in the QL IFP in canine cadavers and assessed the spread of 0.3 versus 0.6 mL kg^−1^ per side of contrast media solution injected through the catheters using computed tomography (CT). The second phase described the outcomes of five dogs undergoing open cholecystectomy or adrenalectomy, in which QL catheters were used for postoperative pain management. CT scan images showed that using a higher volume of injectate resulted in a significantly higher number of vertebral bodies stained by the contrast solution between the QL and psoas minor muscles. Results of the second phase of the study suggest that ropivacaine 0.5% injected uni- or bilaterally every eight hours through QL catheters could be safely implemented in a multimodal analgesic approach during the postoperative period in dogs subjected to abdominal surgeries.

**Abstract:**

This study aimed to describe an ultrasound-guided technique for implanting catheters for local anesthetic administration into the quadratus lumborum (QL) inter-fascial plane in canine cadavers and assessing the spread along the vertebral bodies (VBs) by computed tomography (CT). Phase 1: eight canine cadavers received one catheter per hemiabdomen, followed by injection of contrast media solution [low volume (L_V_) 0.3 mL kg^−1^ or high volume (H_V_) 0.6 mL kg^−1^]. Phase 2: postoperative pain of five dogs was managed by injecting 0.3 mL kg^−1^ of ropivacaine 0.5% through QL catheters every eight hours, up to 72 h after abdominal surgery. Pain was assessed using the Short Form of the Glasgow Composite Measure Pain Scale, and methadone 0.2 mg kg^−1^ was administered intravenously when the pain score was ≥6. The number of VBs stained by the contrast solution between the QL and psoas minor muscles was significantly higher in group H_V_ than group L_V_. The catheter tip was visualized in the retroperitoneal space in 1/16 and 2/10 hemiabdomens in phases 1 and 2, respectively. Rescue analgesia was required in 3/5 dogs during the postoperative period. The QL catheter placement technique appears feasible and may be included in a multimodal analgesic approach for dogs undergoing abdominal surgeries.

## 1. Introduction

The ultrasound (US)-guided quadratus lumborum (QL) block is a locoregional anesthesia technique through which local anesthetic (LA) is injected into the thoracolumbar fascia enveloping the QL muscle. The aim is to desensitize the ventral rami of the spinal nerves and the sympathetic trunk, which are responsible for the somatic and visceral innervation of the abdomen, respectively [1]. The QL block has been reported as an effective technique for abdominal perioperative analgesia in humans [2,3], dogs [4,5], and cats [6]. Recently, cadaveric and clinical studies proposed the inclusion of a caudal QL block in the pain management of dogs undergoing orthopedic hindlimb surgery and orchiectomy [7,8,9].

Garbin et al. evaluated the spread of 0.3 mL kg^−1^ per side of dye-lidocaine solution injected in canine cadavers, performing the QL block with a lateral approach [10]. The injection was performed after positioning the tip of the needle between the lateral aspect of the QL muscle and the transverse process of the first lumbar vertebra (L1). The authors of that study reported a median spread (range) of 4 (3–5) ventral rami of spinal nerves, within the twelfth thoracic (T12) and third lumbar (L3) vertebrae. A consistent spread along the sympathetic trunk within T11 and L3 was also reported. According to the results of a clinical trial performed in female dogs undergoing laparoscopic ovariectomy, this approach resulted in a reduction of perioperative opioid consumption compared to a systemic fentanyl-based protocol [5].

The duration of the postoperative analgesic effect of the QL block in dogs undergoing abdominal surgeries reported in veterinary literature varies between four and 10 h, depending on the LA, volume, and concentration used [4,5]. In the institutions of the authors of this study, the QL block is performed in dogs with severe abdominal pain, poorly responsive to traditional systemic analgesic therapy, both preoperatively and postoperatively. However, to perform this technique safely, dogs need to be deeply sedated or anesthetized, which can be a contraindication. In human medicine, implantation of catheters in the QL inter-fascial plane (IFP) has been described to extend the duration of effect of a single-shot block in the postoperative period [11,12,13]. Recently, the insertion of catheters into the transversus abdominis plane (TAP) and between the QL and psoas minor (PM) muscles in dogs has been reported in two case series [14,15]. In both reports, administration of bupivacaine 0.5% through the catheters every six or 12 h resulted in a reduction in pain scores in dogs after different abdominal surgeries or in the presence of pancreatitis.

The aim of this study was to (1) describe an US-guided technique for placing catheters for LA administration in the QL IFP in canine cadavers, (2) assess the spread of two different volumes (0.3 versus 0.6 mL kg^−1^) per side of contrast media solution injected through the catheters using computed tomography (CT), and (3) describe the outcomes of five dogs undergoing different abdominal surgeries, in which catheters bilaterally implanted in the QL IFP were included in a multimodal postoperative analgesic plan. We hypothesized that this technique is feasible in dogs and that using a higher volume of injectate would result in greater spread along the vertebral bodies (VBs).

## 2. Materials and Methods

This study was divided into two phases. In the first phase, eight canine cadavers were used to describe the technique and assess the spread of the contrast media solution by computed tomography (CT). Ethical approval was obtained from the University of Liege, Belgium, approval for animal experimentation (Commission d’éthique animale; n. 2647). In the second phase, we report the use of bilateral QL catheters in five client-owned dogs for ropivacaine intermittent administration for postoperative analgesia after different open surgeries. Each owner signed an informed written consent and agreed that data from their animals could be used for the purpose of this study.

### 2.1. First Phase

In the first phase of this study, we included eight canine cadavers intended for hands-on training in general surgery for students at the Faculty of Veterinary Medicine of the University of Liege. Dogs were euthanized for reasons unrelated to this study. Exclusion criteria comprised the presence of lesions on skin or muscles of the thoracolumbar area, as well as alterations in the conformation and number of vertebrae between T8 and L7.

#### 2.1.1. US-Guided Catheter Placement Technique

**Figure 1:**
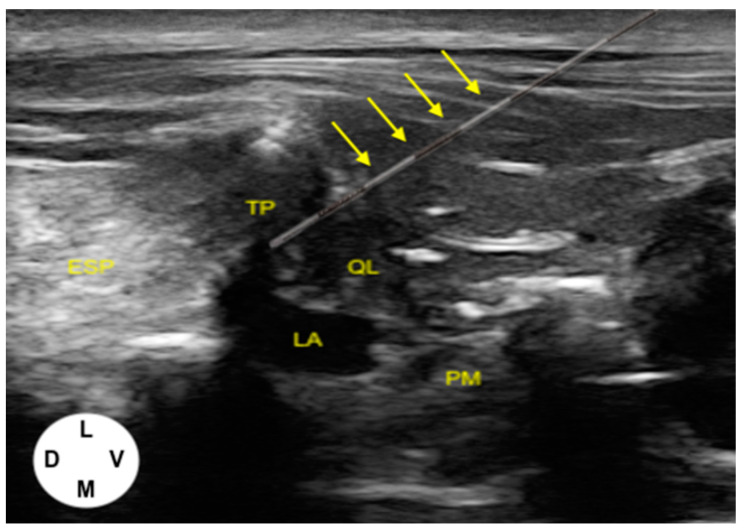
Post-injection ultrasound image of the anatomical structures and needle trajectory (yellow arrows) to perform the lateral quadratus lumborum (QL) block: QL, quadratus lumborum muscle; PM, psoas minor muscle; TP, transverse process of L1; ESP, erector spinae muscles; LA, local anesthetic.

Cadavers were positioned in either lateral recumbency, and the hair on the dorso-lateral thoracolumbar area of both hemiabdomens was clipped. Complete sets for epidural anesthesia (Perifix^®^ Complete Set, B Braun, Melsungen, Germany) with a Tuohy needle (1.30 × 80 mm, 18 G), catheter (0.85 × 0.45 × 1000 mm, 20 G), filter, and a self-adhesive securement device were used in this study. The lateral approach described for the US-guided QL block [10] was used to place all catheters with a 14 MHz high-frequency linear transducer (L7HD3VET; Clarius Mobile Health, Vancouver, BC, Canada), connected to a touchscreen tablet (iPad Air, fifth generation; Apple, Cupertino, CA, USA). Implantations of catheters were performed by the same investigator (M.D.), assisted by a second one (C.T.). The transverse process of L1, the QL, and the erector spinae (ESP) muscles were sonographically visualized (Figure 1). The needle was introduced with an in-plane technique in a ventrolateral-to-mediodorsal direction and advanced towards the ventral aspect of the transverse process of L1, with the bevel of the needle directed dorsally. Once the needle passed through the thoracolumbar fascia and came into contact with the transverse process, the bevel was rotated 180° in a ventral direction, and a bolus of 0.05 mL kg^−1^ of saline solution (Mini-Plasco NaCl; B Braun, Germany) was injected. Confirmation of correct positioning of the needle was obtained by visualization of the hydrodissection between the dorsal aspect of QL muscle and the ventral aspect of the transverse process of L1 (Figure 1). Then, the catheter was inserted through the needle and advanced 3–4 cm into the IFP. The needle was removed, and the catheter was fixed to the skin using a 3-0 suture (Ethilon; Ethicon, Raritan, NJ, USA), with a finger-trap pattern. The catheter was cut approximately 15 cm from the insertion point of the skin and secured with the self-adhesive device. Time to perform the technique, considered the interval between the start of the US scanning and withdrawal of the Tuohy needle, was recorded. The same technique was performed on both sides.

#### 2.1.2. Tomographic Study

After implantation of both catheters, CT scans of the region between T8 and L7 were performed using a 64-slice multidetector scanner (Somatom Confidence 64, Siemens, Erlangen, Germany). Cadavers were positioned in sternal recumbency, with their forearms and hindlimbs extended. Each cadaver received one injection per hemiabdomen, performed through the catheter, either with 0.3 (group L_V_) or 0.6 mL kg^−1^ (group H_V_) of injectate. The solution for injection was prepared by adding 25 mL of ioexhol radiographic contrast media (Ultravist 300, Bayer, Berlin, Germany) to 500 mL of saline solution (Vetivex^®^ 9 mg mL^−1^, Dechra Veterinary Products, Belgium). Right and left-sided injections were randomized using an online software software program (www.random.org, accessed on 1 January 2024). A second investigator (C.T.) performed the injections by hand, maintaining the speed as consistently as possible but not specifically controlled. Acquisition parameters used depended on the size of the dog (ranges: 100–140 kV, 250–300 mAs, and pitch factor = 0.9). Scan tube current was modulated by automatic exposure control (Care Dose, Siemens Medical Solutions, International). Image data sets were reconstructed using parameters of 120 mm field of view, 512 × 512 matrix, 1 mm slice thickness, and the Br34 reconstruction algorithm (window level 45 and window width 410). A three-dimensional reconstruction of the CT images was performed using (Syngo.via (VB60A_HF06), MM Reading, Siemens Healthineers, Erlangen, Germany), and a board-certified radiologist (G.B.) blinded to group allocation assessed the location and the distribution of the contrast medium by counting the number of VBs. Visualization of radiographic contrast between the QL and PM muscles (spinal nerve area), as well as ventromedially to PM muscle (sympathetic trunk area), was recorded and compared between the groups. The location of the catheter insertion site into the skin was also recorded for each hemiabdomen.

### 2.2. Second Phase

#### 2.2.1. Cases Presentation

Five dogs of different breeds and American Society of Anesthesiologists (ASA) statuses, undergoing various open adrenalectomy or cholecystectomy, were recruited in the second phase of the study. Demographic and medical data are reported in Table 1. Procedures were performed at the Veterinary Teaching Hospital of the University of Liege between May 2024 and July 2024. Prior to anesthesia, each dog underwent a complete physical examination, including a body condition score (BCS) on a nine-point scale evaluation [16], and blood test screening (hematology, serum biochemistry, coagulation times). Physical examinations were unremarkable for all dogs, except for Case 3. A moderate-severe left apical systolic murmur was detected. An echocardiography was performed and revealed stage B1 mitral valve endocardiosis. The anesthetic protocol was at the discretion of the anesthesiologist in charge of each case. However, all dogs received intraoperative fluid therapy with lactated Ringer’s solution at 3–5 mL kg^−1^ h^−1^ and a preoperative QL block performed with 0.3 mL kg^−1^ of ropivacaine 0.5% per side [10].

#### 2.2.2. Catheter Placement and Postoperative Management

Catheters were implanted bilaterally at the end of the surgery, following the technique described in the first phase of the study. The region was clipped and scrubbed, and the procedure was performed aseptically. Catheters were fixed to the skin as previously described and then anchored to the adhesive pad provided in the kit for epidural catheters. The anti-bacterial filters were connected and then sutured to the skin. A semi-occlusive dressing and a tubular bandage (TG, Lohmann and Rauscher, Neuwied, Germany) were used to protect the catheters. The dogs were then moved to the CT scan room. A volume of 0.1 mL kg^−1^ of contrast media solution, divided in two aliquots, was injected through each catheter, and a CT scan was performed after each injection. Once the catheter tip or the spread around the QL muscle was visualized, the second aliquot was injected, and a second CT scan was performed to confirm the spread in the same target areas evaluated in the first phase of the study. At the end of the procedure, anesthesia was discontinued, and tracheal extubation was performed once the swallowing reflex was restored. Dogs were then transferred to the Intensive Care Unit, and a standardized pain management protocol was applied, as follows: Pain was assessed every hour using the Short Form of the Glasgow Composite Measure Pain Scale (SF-GCMPS) [17] starting one hour after extubation (T_E_) until the pain score was ≥6 (T0). A volume of 0.3 mL kg^−1^ of ropivacaine 0.5% was injected through the catheters at T0 and then every eight hours up to 72 h. Pain assessment was repeated every four hours as of T0, or more frequently if the dogs were deemed uncomfortable or unsettled. The evaluations were carried out by the critical care clinician present in clinics at the time of assessment. In cases of pain score ≥6/24, methadone 0.2 mg kg^−1^ was administered intravenously (IV). Hospitalization time, pain scores, and number of rescue methadone administrations during the postoperative period were recorded.

### 2.3. Statistical Analysis

The number of cadavers needed for the study was calculated based on the spread of contrast media solution. The alpha error was set at 0.05 and the power at 80%. Considering a mean ± standard deviation (SD) of VBs involved in the spread to be 4 ± 1 in group L_V_ and six in group H_V_ [10], the number of dogs required to assess a significant difference between the two groups was calculated to be four. This number was then increased to eight to ensure sufficient data to perform statistics and to account for any potential variability in procedural execution. The normality of data distribution was evaluated with a D’Agostino and Pearson test, and results are expressed as mean ± SD or median (range). Prism Version 9.0 (GraphPad Software Inc., San Diego, CA, USA) was used to analyze data. A Fisher’s exact test was used to compare the number of VBs stained between sides (right and left), and a Mann-Whitney test was used to compare the number of VBs involved in the spread between H_V_ and L_V_. A Student’s T-test was used to compare the time to position the catheter for each side. Data are reported as numbers of cases (n/n) and percentages (%). A *p*-value < 0.05 was considered statistically significant.

## 3. Results

### 3.1. First Phase

The mean ± SD weight of the cadavers was 19.1 ± 6.7 kg, and the median (range) body condition score (BCS) was 5 (4–6) out of 9. Breeds, sex, weight, and BCS are reported in Table 2.

#### 3.1.1. US-Guided Catheter Placement

The catheter placement technique was completed in all 16 hemiabdomens. Time to perform the technique was 255 ± 65.8 s on the right hemiabdomen and 273 ± 81.5 s on the left hemiabdomen (*p* = 0.6). The insertion point of the catheter into the skin was localized at the level of L2, L3, and L4 in 12.5%, 50%, and 37.5% of cases, respectively.

#### 3.1.2. Tomographic Study

Contrast media solution was injected in all hemiabdomens, except one in group Hv. In that particular case, unusually high resistance to injection was noted, and the catheter tip was then tomographically visualized in the retroperitoneal space at the level of T13. In all other cases, it was not possible to clearly visualize the catheter tip location because of the presence of the contrast medium effacing the border of the catheter tip.

**Figure 2:**
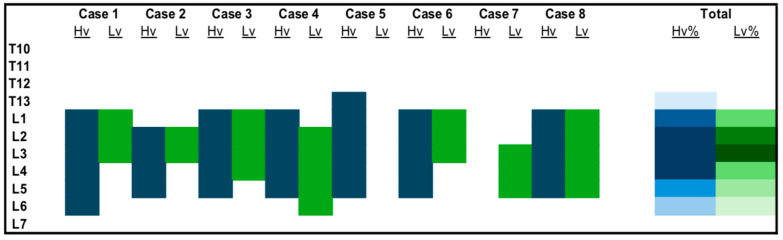
Stain of vertebral bodies (VBs) between the quadratus lumborum (QL) and psoas minor (PM) solution muscles in the cadavers included in this study after high volume (H_V_) or low volume (L_V_) of contrast media solution. For the total amount, the color intensity is indicative of the success rate, with dark shading indicating a higher success rate.

A contrast solution was visualized between the QL and PM muscles in 7/8 hemiabdomens in group L_V_ and 6/7 hemiabdomens in group H_V_. At this level, the number of VBs stained by the contrast was significantly higher (*p* = 0.01) in group H_V_, 5 (4–6), compared to L_V_, 3.5 (0–5) (Figure 2). The sympathetic trunk area was involved by the spread in 3/8 and 6/7 hemiabdomens, in groups L_V_ and H_V_, respectively. At this level, the number of VBs covered by the spread did not result significantly different between the two groups (*p* = 0.08): 0 (0–5) for group L_V_ and 4 (0–5) for group H_V_.

**Figure 3:**
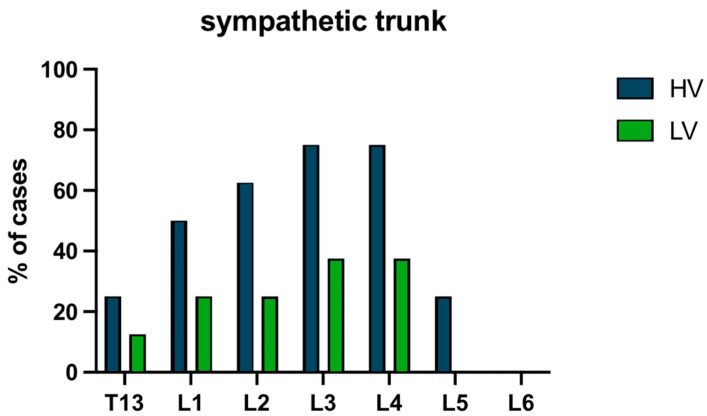
Distribution of contrast solution ventromedially to the psoas minor muscle (sympathetic trunk area) at the level of the different vertebral bodies. Data are expressed in percentage (%).

The spread of contrast between the QL and PM muscles in group L_V_ was mostly found at the levels of L3 (87.5%), and L2 (75%), slightly less at L1 and L4 (50%), L5 (37.5%), and L6 (25%). In group H_V_, spread of contrast media solution was consistently detected between QL and PM muscles at the levels L2, L3, and L4 (87.5%), less frequently at L1 (75%) and L5 (62.5%), and finally at L6 (25%) and T13 (12.5%) (Figure 2). The spread of injectate was visualized in the sympathetic trunk area between T13 and L4 (37.5% of cases at L3 and L4, 25% at L1 and L2, and 12.5% of cases at T13) in group L_V_, and between T13 and L5 (75% at L3 and L4, 62.5% at L2, 50% at L1, 62.5% at L2, and 25% at L5 and T13) in group H_V_ (Figure 3).

**Figure 4:**
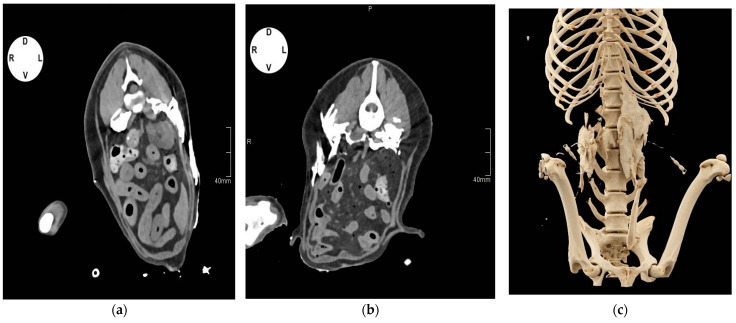
Computed tomographic images in transverse plane with soft tissue window of canine cadavers after injection of different volumes of contrast media solution through catheters implanted in the quadratus lumborum (QL) plane. (**a**) Transverse image at L3: contrast can be visualized in the transversus abdominis plane (TAP), between the QL and psoas minor (PM) muscles, and subcutaneously. (**b**) Transverse image at L3: Contrast can be observed between the QL and PM muscles, ventromedially to the PM muscle, in the TAP and ESP plane. (**c**) Volume-rendered three-dimensional reconstruction image of the thoracolumbar area between T12 and L4 vertebrae showing distribution of the contrast and catheter positioning.

Erratic distribution of contrast medium was observed in both groups in the retroperitoneal space in 37.5% of cases in group H_V_ and 50% in group L_V_, and in the ESP plane in 28.5% of cases in group H_V_ and 25% in group L_V_. Contrast was found also in the TAP (37.5% of cases in group H_V_ and 50% in group L_V_) and abdominal subcutaneous tissue (28.5% of cases in group H_V_ and 37.5% in group L_V_), mostly between L2 and L4. No contrast medium was visualized in the epidural space, peritoneal, or intrathoracic cavity (Figure 4a,b).

### 3.2. Second Phase

Five dogs were included in the second phase of the study. The median (range) age and weight of the study were 9 (6–11) years old and 18.3 (4.6–29) kg, respectively. Surgery and anesthesia concluded uneventfully in all cases. Data regarding type of surgery, anesthetic protocol, intra- and postoperative medications, duration of anesthesia and surgery, and time between T_E_ and T0 are reported in Table 3.

**Figure 5:**
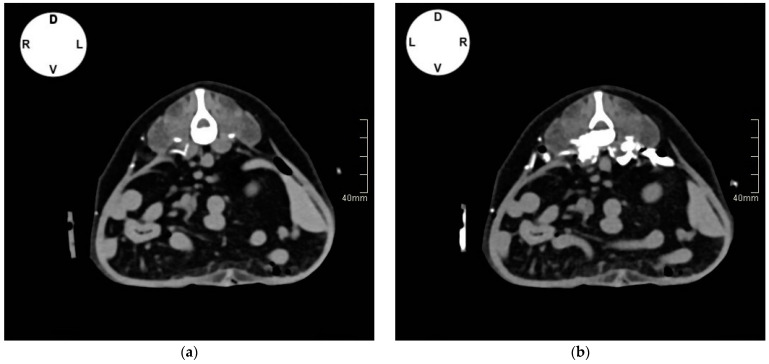
(**a**) Computed tomographic (CT) images in transverse plane (L2) with soft tissue window of Case 3 after injection of contrast media solution through catheters implanted in the quadratus lumborum (QL) plane. (**a**) Catheter tip and contrast media can be visualized between the QL and psoas minor (PM) muscles on the right side after injection of 0.5 mL of solution. (**b**) Spread of 0.05 mL kg^−1^ of contrast media between the QL and psoas minor (PM) muscles, inside and ventromedially the PM muscle, between the transversalis and thoracolumbar fasciae.

The QL catheter placement technique was completed in all five dogs. After the first injection of contrast, the catheter’s tip was visualized dorsally to the QL muscle or between the QL and PM muscles in all cases (at the level of L1 in Cases 1 and 5, L2 in Cases 2,3, L3 in Case 4) except for one catheter in Case 1 and one in Case 5, where the catheter tip was found, respectively, in the left- and right-side retroperitoneal space. In both cases, the mispositioned catheter was withdrawn, and injections of ropivacaine were performed only on the controlateral side. The CT scan images taken after injecting the second aliquot of contrast showed spread of solution in the target areas in all remaining cases. (Figure 5a,b).

**Figure 6:**
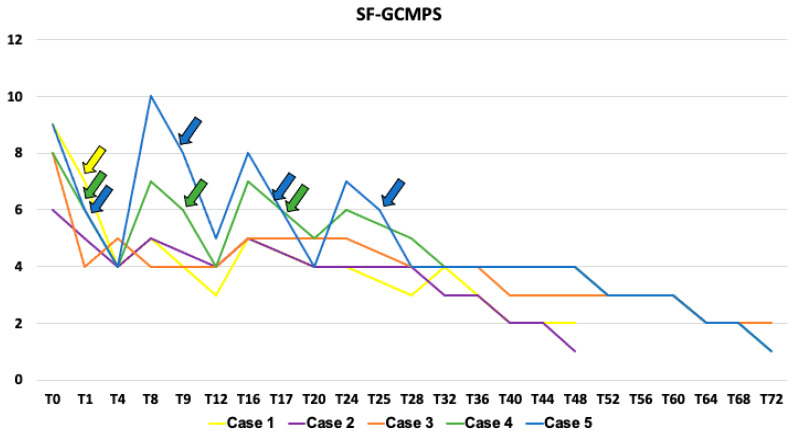
Postoperative pain scores at different time points, using the short-form Glasgow Composite Measure Pain Scale (SF-GCMPS) in the five dogs enrolled in the study. Arrows: administration of methadone 0.2 mg kg^−1^ IV as rescue analgesia. Arrow colors correspond to the cases receiving rescue analgesia, matching the line colors in the graph.

Methadone 0.2 mg kg^−1^ was administered IV once in Case 1, three times in Case 4, and four times in Case 5 during the first 24 h after surgery (Figure 6).

No rescue analgesia was required in the remaining postoperative period. An opioid-free pain management was achieved in Case 2 and Case 3 for 48 and 72 h, respectively. No complications associated with the use of QL catheters, such as infections, motor impairment, or systemic side effects to ropivacaine, were recorded during the study period. Catheters were removed 48 and 72 h after surgery in Cases 1 and 2, and 3 to 5, respectively.

## 4. Discussion

The first part of this study aimed to describe an US-guided technique to implant catheters for bilateral LA administration in the QL IFP in canine cadavers and to compare the spread of 0.3 versus 0.6 mL kg^−1^ of contrast media solution along the VBs. The second part described the outcomes of five dogs, in which the injection of ropivacaine 0.5% through QL catheters was implemented in a multimodal analgesic regimen for postoperative abdominal pain. The use of a larger volume resulted in a significantly higher number of VBs affected by the spread only between the QL and PM muscles, but not in the sympathetic trunk area. The case series demonstrated the clinical feasibility of the technique and the potential of an intermittent LA injection through QL catheters for implementation in the management of post-laparotomy pain in dogs.

The first phase demonstrated the feasibility of the US-guided catheter placement technique described in this study. A previous case series reported the use of the trans-muscular approach [18] to implant catheters between the QL and PM muscles [15]. We decided to opt for the lateral approach [10], directing the tip of the needle towards the latero-ventral aspect of the transverse process of L1, because the visualization of the plane between QL and PM muscles is not always achievable in both small and large breed dogs [10,19]. The time required to perform the technique was acceptable, suggesting its potential for routine practice with adequate training. However, procedures were performed by two anesthetists experienced in US-guided locoregional anesthesia, which may limit generalization of the results.

Garbin et al. injected 0.3 mL kg^−1^ of dye-lidocaine solution in canine cadavers, observing a median spread involving four (3–5) ventral rami of spinal nerves between T13 and L3 after dissection [9]. Results obtained in this study, using the same volume of contrast media solution, are similar. Increasing the volume to 0.6 mL kg^−1^ resulted in a significantly higher number of VBs involved by the spread but not in diffusion towards those between T9 and T13, as already reported in veterinary literature [10,18,19,20,21,22]. In addition, contrast media solution was observed in the sympathetic trunk area in a higher number of hemiabdomens in group H_V_ in comparison with group L_V_. These findings are in contrast with results obtained by Garbin et al., who reported a spread to the sympathetic trunk between T11 and L2 after injecting 0.3 mL kg^−1^ of dye-lidocaine solution [10]. However, it is important to note that our study did not involve anatomical dissection of cadavers. This limitation is significant because the distribution of contrast media often does not align with the staining of nerves [21]. Our results suggest that higher volumes of LA could result in more extensive desensitization of the abdomen. However, clinicians should be aware that increasing the volume of injectate would result in dilution of LA and therefore in decreased analgesic effect duration in the postoperative period [5].

The tomographic study revealed a caudally directed spread, aligning with previous findings [21,22]. Viscasillas et al. hypothesized that this finding could be a consequence of the needle trajectory, which was inserted in a caudal direction, from dorsolateral to ventromedial [22]. Despite advancing the needle in the opposite direction, the catheter tip might have turned caudally during advancement. Additionally, in the present study, the insertion point of the catheter into the skin was positioned caudally to L1 in all cases, likely due to anatomical differences in rib conformation. Contrast media distribution was most consistently observed at the level of VBs between L2 and L4, suggesting a catheter tip position caudal to L1, though the exact location was not confirmed.

CT scans showed contrast media spread reaching L5 and L6 in both groups. Otero et al. (2024) described a caudal approach to the QL block, reporting spread towards the lumbar plexus with a 0.3 mL kg⁻^1^ dye solution injected at L6 [7]. In clinical trials, tonic muscle function was maintained with lidocaine 2% and ropivacaine 0.5% without any postoperative motor deficits [7,8,9]. Similarly, in our clinical case series, no dogs experienced hindlimb weakness secondary to possible motor impairment. However, in the second phase of the study, a CT scan was used only to confirm catheter positioning and not to assess the spread of the solution along the VBs.

Contrast media was frequently observed in the retroperitoneal space, possibly due to the catheter tip perforating the transversalis fascia. Catheters were advanced 3–4 cm into the QL IFP, similarly to Camargo Fontanela et al., who confirmed final positioning under US guidance, by injecting 1 mL of bupivacaine and observing diffusion into the IFP. However, despite recording an analgesic effect, the authors of that study could not rule out catheter displacement [15]. In this study, contrast injection was performed after repositioning dogs from lateral to sternal recumbency, simulating a likely natural movement of a dog during the postoperative period. It cannot be ruled out that changes in recumbency and abdominal muscle tension might lead to catheter tip dislodgment during the postoperative period. Further research is necessary to investigate and refine this technique, as well as to assess accurate catheter positioning in the QL IFP.

Diffusion of contrast in the TAP and subcutaneous tissues may be produced by leakage of solution from the insertion site, potentially caused by the difference in size between the Tuohy needle (18 G) and the catheter (20 G). In human medicine, LA leakage from catheters is a common complication that can result in block failure and inadequate analgesia [23,24]. In our study, the erratic spread of contrast to the TAP and subcutaneous tissue could explain the reduced diffusion to the sympathetic trunk area, recorded in group L_V_. This information could be crucial when selecting the appropriate needle and catheter sizes, especially in small-breed dogs. However, dislodgement of the catheter tip from the QL plane due to the change in recumbency of the cadavers during the procedure cannot be ruled out. Catheter dislodgment and secondary block failure are well-documented complications in humans [25]. Further research assessing catheter displacement or dislodgement rates is warranted in veterinary medicine [26].

Erratic spread of contrast in the epaxial compartment could be attributed to perforation of the thoracolumbar fascia, as previously hypothesized by Alaman et al. [20]. According to the technique described in this study, the needle was initially advanced with the bevel dorsally directed to improve its sonographic visualization and then rotated to allow the catheter to advance ventrally to the transverse process of L1. It is possible that the 180° rotation of the Tuohy needle bevel could have resulted in inadvertent perforation or laceration of the thoracolumbar fascia.

In the second phase of this study, catheters implanted in the QL IFP were used in five dogs undergoing open cholecystectomy or adrenalectomy. Dogs were included in the study based on the expected pain levels caused by the surgery. Cholecystectomy is known to cause significant postoperative pain, warranting effective analgesic management, such as the use of an epidural catheter or a QL block [1,27,28]. Based on the authors’ clinical experience, open adrenalectomy can result in moderate-severe postoperative abdominal pain, requiring administration of different systemic analgesics in IV infusion, such as opioids, ketamine, and lidocaine [29].

Based on the pain scores, ropivacaine 0.5% administered via QL block and through catheters three times daily could be a valid integration into a multimodal analgesic regimen for postoperative abdominal pain in dogs. According to the design of this study, pain assessments were performed at four hour intervals to ensure a pain evaluation exactly halfway between two administrations of LA. Injections of ropivacaine 0.5% reduced pain scores at the four-hour post-administration assessment. However, in cases where pain scores remained ≥6 on the SF-GCMPS, rescue analgesia consisting of methadone 0.2 mg kg^−1^ IV was administered. This occurred in Case 1 (once), Case 4 (three times), and Case 5 (four times). Cases 2 and 3 did not require additional methadone within the initial 48–72 h postoperatively. It cannot be ruled out that the need for rescue analgesia was due to potential dislodgment of the catheter, resulting in inadequate spread of LA to the target areas of the QL block. Moreover, cadaveric studies have shown that QLB, regardless of the approach used, often fails to produce adequate spread to the last thoracic ventral rami of the spinal nerves [10,18,19,20,21,22]. As a result, desensitization of the cranial portion of the abdomen may be incomplete, potentially contributing to the need for rescue analgesia.

Ropivacaine was administered unilaterally due to catheter misplacement in the retroperitoneal space, in Cases 1 and 5, where rescue analgesia was required once and four times, respectively. The variation in outcomes between these two cases may be attributed to the fact that the catheter that remained in place in Case 1 was on the same side as the adrenalectomy, where most visceral manipulation occurred during surgery.

Our findings align with reports in human medicine regarding the effectiveness of QL catheters in reducing postoperative opioid use and improving pain management [11,12,13]. In the case series by Camargo Fontanela et al., three dogs with severe abdominal pain were effectively managed with intermittent administration of 0.3 mL kg^−1^ per side of bupivacaine 0.5% twice daily [15]. The authors chose to use the same volume of ropivacaine 0.5%. This decision was taken based on their clinical preference in using low volume-high concentration solutions of LA when performing IFP blocks [5,30] to avoid excessive dilution and secondary reduction of analgesic effect duration. According to previous research, the analgesic duration of ropivacaine 0.5% administered via QL block after canine laparoscopic ovariectomy was approximately 10 h [5]. In this case series, we chose to administer ropivacaine 0.5% every eight hours to ensure analgesic effect before the LA began to wear off.

There are some limitations to be considered when interpreting these results. In the first phase of the study, injections of contrast media solution were performed in thawed canine cadavers. Density and viscosity of the contrast solution used in this study may be different from those of LA. In addition, the spread of solution observed in cadavers does not necessarily reflect the one from live animals, due to different biophysical properties of tissues, such as fasciae integrity and membrane permeability. Anatomical dissections were not performed because the canine cadavers were meant to be utilized for practical teaching purposes after the end of our experiment. CT scan images provide information on the diffusion pattern of the injectate and not on which structures are effectively stained [21], therefore comparisons with previous studies where cadavers were dissected might be complicated. Additionally, dissections could have been helpful to assess the position of the catheter tip. The small sample size, lack of a control group, and influence of different analgesic drugs used in the perioperative period may pose difficulties in drawing solid conclusions on the analgesic efficacy of the technique presented in this study. Additionally, the dogs underwent different types of surgery, which introduces further variability. Finally, clinicians in charge of postoperative pain assessment were not blinded to the treatment, which could have biased the results.

## 5. Conclusions

US-guided catheter placement in the QL IFP appears feasible and may enhance postoperative analgesic management in dogs undergoing abdominal surgeries. This technique could reduce the systemic opioid consumption and improve pain management protocols. Further research is warranted to optimize the technique for clinical use, and larger sample sizes and standardized protocols are necessary to validate these findings. Clinicians should be aware of potential catheter misplacement, dislodgement, or leakage of LA from the insertion site, which could reduce the analgesic effect or lead to block failure.

## Figures and Tables

**Table 1:** Demographic and medical data of the five dogs included in the study. ASA, American Society of Anesthesiologists; BCS, body condition score (nine-point scale evaluation); F, female; M, male; MC, male castrated.

	Case 1	Case 2	Case 3	Case 4	Case 5
Breed	Siberian Husky	Beagle	Chihuahua	Brittany Spaniel	Mixed breed
Sex	F	M	M	MC	MC
Age (years)	6	8	11	9	11
Weight (kg)	23.5	29	4.6	18.3	13
BCS	5/9	6/9	5/9	5/9	4/9
ASA status	3	2	4	3	4
Condition	Right-sided cortisol-secreting adenocarcinoma	Left-sided non-secreting adrenal mass	Emphysematous cholecystitis, pancreatitis	Right-sided phaeochromocytoma	Gallbladder mucocele, pancreatitis

**Table 2:** Breed, sex, weight, and BCS (nine-point scale evaluation) of the canine cadavers used in the study.

Breed	Sex	Weight	BCS (Out of 9)
English Setter	Male castred	23	5
Border Collie	Female	17	4
Mixed breed	Male castred	22	6
Mixed breed	Female	13	5
American Amstaff	Male castred	22	5
Border Collie	Male castred	25	6
Jack Russell	Male castred	6	4
Siberian husky	Female	25	5

**Table 3:** Data regarding type of surgery, anesthetic protocol, intra- and postoperative medications, duration of anesthesia and surgery, and time between T_E_ and T0. T_E,_ one hour after extubation; T0, first administration of ropivacaine through catheters (pain score ≥ 6 out of 24).

	Case 1	Case 2	Case 3	Case 4	Case 5
Surgery	Right-sided adrenalectomy	Left-sided adrenalectomy	Cholecystectomy	Right-sided adrenalectomy	Cholecystectomy
Premedication	Dexmedetomidine 1 μg kg^−1^, methadone 0.2 mg kg^−1^ IV	Dexmedetomidine CRI 1 μg kg^−1^ h^−1^, methadone 0.2 mg kg^−1^ IV	Maropitant 1 mg kg^−1^, methadone 0.2 mg kg^−1^ IV	Acepromazine 10 mcg kg^−1^ IM, methadone 0.2 mg kg^−1^ IV	Maropitant 1 mg kg^−1^, methadone 0.2 mg kg^−1^ IV
Induction	Propofol 2 mg kg^−1^	Propofol 2 mg kg^−1^ IV	Ketamine 1 mg kg^−1^, propofol 1 mg kg^−1^ IV	Propofol 2 mg kg^−1^ IV	Midazolam 0.2 mg kg^−1^ Propofol 1.5 mg kg^−1^ IV
Maintenance	Isoflurane in an oxygen/air mixture	Isoflurane in an oxygen/air mixture	Isoflurane in an oxygen/air mixture	Isoflurane in an oxygen/air mixture	Isoflurane in an oxygen/air mixture
Intraoperative medication	Dexmedetomidine CRI 1 μg kg^−1^ h^−1^ IV	-	Fentanyl infusion 5–10 μg kg^−1^ h^−1^ IV	Fentanyl infusion 2–5 μg kg^−1^ h^−1^ IV	Ketamine 1 mg kg^−1^ IV
Postoperative medication	Prednisolone 0.5 mg kg^−1^ IV BID, cefazoline 20 mg kg^−1^ IV TID, and trazodone 4 mg kg^−1^ PO TID	Meloxicam 0.2 mg kg^−1^ IV SID, cefazoline 20 mg kg^−1^ IV TID	Meloxicam 0.2 mg kg^−1^ IV SID, amoxicilline clavulanic acid 20 mg kg^−1^ TID maropitant 1 mg kg^−1^ SID, ondasetron 0.5 mg kg^−1^ PO BID, pimobendan 0.5 mg kg^−1^ PO BID	Meloxicam 0.2 mg kg^−1^ IV SID, cefazoline 20 mg kg^−1^ IV TID, trazodone 4 mg kg^−1^ TID	Meloxicam 0.2 mg kg^−1^ IV SID, amoxicilline clavulanic acid 20 mg kg^−1^ TID, maropitant 1 mg kg^−1^ SID
Duration of anesthesia (minutes)	360	255	90	240	120
Duration of surgery (minutes)	300	210	60	180	65
T_E_ − T0 (minutes)	60	180	300	180	300
Hospitalization time (hours)	48	48	72	72	72

## Data Availability

Data supporting the reported results can be sent to anyone interested by contacting the corresponding author.
